# Efficacy and influencing factors of cervical perivascular sympathectomy in children with cerebral palsy

**DOI:** 10.1007/s00381-023-06191-w

**Published:** 2023-10-23

**Authors:** Junjie Wu, Baofeng Yan, Nurehemaiti Mutalifu, Qi Guan, Chao Bai, Jianglong Li, Xinping Luan

**Affiliations:** Cerebral Palsy Center in Neurosurgery, Second Affiliated Hospital of Xinjiang Medical University, Nanhu North Road, Shuimogou District, Urumqi, Xinjiang 830063 China

**Keywords:** Cerebral palsy, Cervical perivascular sympathectomy, Communication function, Salivation, Retrospective cohort studies

## Abstract

**Background:**

There is a lack of research to determine the efficacy of cervical perivascular sympathectomy (CPVS) in children with cerebral palsy (CP).

**Objective:**

This study aimed to evaluate the efficacy of CPVS in children with CP and analyze the associated influential factors.

**Methods:**

Using the method of retrospective cohort studies, children who underwent CPVS were included in the CPVS group, whereas those who underwent selective posterior rhizotomy (SPR) were included in the SPR group. The Communication Function Classification System (CFCS) and Teacher Drooling Scale (TDS) were used to evaluate the communication function and salivation in the two groups before and 12 months after surgery and compare the surgical efficiency between the two groups, and the factors affecting the efficacy were screened by binary logistic regression.

**Results:**

The study included 406 patients, 202 in the CPVS group and 204 in the SPR group. No significant differences were observed in the baseline characteristics (*p* > 0.05). The surgical efficacy of the CPVS group (47.01%) was significantly higher than that in the SPR group (9.81%) (*χ*^*2*^ = 71.08, *p* < 0.001). Binary logic regression analysis showed that preterm birth and Gross Motor Function Classification System (GMFCS) grade were influencing factors of surgical efficacy. Eighteen patients developed postoperative complications.

**Conclusion:**

CPVS is a safe and effective surgery for cerebral palsy. Preterm birth and GMFCS grade are independent factors affecting the efficacy of surgery.

## Introduction

Cerebral palsy (CP) is a group of permanent motor and postural developmental impairments because of non-progressive damage to the developing fetal or infant brain, resulting in motor deficits [1]. The characteristic features of CP are hypotonia, spasticity, weakness, and involuntary movements (with dyskinesia being the commonest symptom), either alone or in combination [2]. In addition to the description of motor characteristics, CP may also have communication disorders and salivation. A communication disorder is observed in approximately 30–80% of CP [3]; about 22 to 44% have salivation [4, 5]. Communication disorders and salivation increase the burden on the parents of CP [6] and make it difficult for CP to participate in education and integrate into society, resulting in low self-esteem and a serious decline in overall quality of life [7].

CP has a current prevalence of 2–3‰ [8]. Cervical perivascular sympathectomy (CPVS), selective posterior rhizotomy (SPR), and rehabilitation are the primary treatment options for CP. CPVS, which was first used in children with neurological disorders, can prevent vascular malformation progression in children with moyamoya disease (MMD) [9, 10]. However, due to its questionable efficacy, it is not the preferred surgical treatment for MMD [11].

CPVS, used for treating CP since 1991, effectively relieves and salivation communication function in children with CP [12, 13]. Despite the role of CPVS in improving communication function and salivation in children with CP, the growth and developmental factors influencing surgical efficacy in children with CP cannot be excluded owing to the lack of a control group. Therefore, there is a lack of evidence on the efficacy of CPVS [14]. The purpose of our study is to explore the efficacy of CPVS in children with CP and to clarify the relevant factors affecting the efficacy of surgery to help clinicians predict the efficacy of surgery in advance and formulate more reasonable treatment plans according to the condition of children with CP.

## Materials and methods

### Sample sources

This study initially recruited children visiting the CP Center in Neurosurgery at the Second Affiliated Hospital of Xinjiang Medical University between January 2018 and January 2022. The inclusion criteria were as follows: (i) patients who met the diagnostic and staging criteria of the internationally recognized CP diagnostic criteria [15]; (ii) with varying degrees of communication dysfunction or salivation; (iii) patients aged 2–18 years. The exclusion criteria were as follows: (i) patients with abnormal behavior, severe mental retardation; (ii) patients with other neurological diseases, such as hydrocephalus, stroke, and meningitis that could lead to communication disorder or salivation; (iii) CP patients with other disorders that can affect communication or salivation; (iv) patients who could not undergo a clinical examination and surgery general anesthesia; (v) patients’ families refusing to follow-up. The Medical Ethics Committee of the Second Affiliated Hospital of Xinjiang Medical University approved this study (20160302-14). Informed consent for the surgical procedures and all treatment measures was obtained from the patients and their families.

### Surgical methods

#### CPVS

**Fig. 1:**
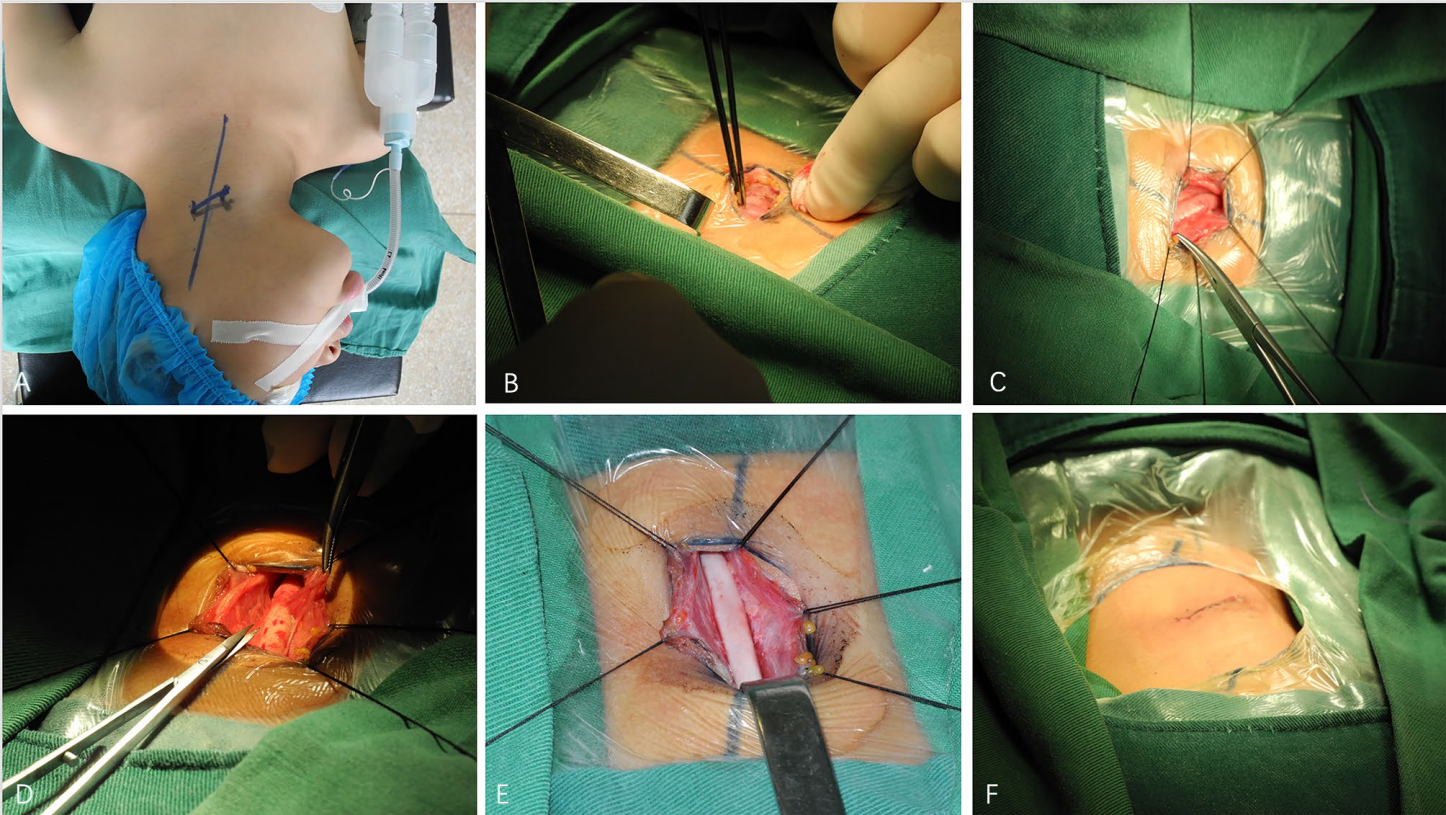
Intraoperative photographs of CPVS. **A** Surgical incision marking. **B** A layer-by-layer skin incision was performed with subcutaneous longitudinal separation of the platysma. **C** The common carotid artery was exposed. **D** Exfoliation of the carotid adventitia. **E** Peeled carotid artery

After intravenous general anesthesia with tracheal intubation, the patient was placed in a supine position with shoulder pads and the head tilted back to one side. After routine disinfection, a transverse incision measuring approximately 3.0 cm in length was made 1 cm below the thyroid cartilage (medial border of the lateral sternocleidomastoid muscle). Following this, a layer-by-layer skin incision was performed with subcutaneous longitudinal separation of the platysma. Subsequently, the superficial layer of cervical fascia was incised, the sternocleidomastoid muscle was pulled laterally, and the thyrohyoid muscle was drawn medially. Following this, the carotid artery was exposed by incising the carotid sheath. In addition, the common carotid artery was dissociated from the carotid sheath to protect the jugular vein and the vagus nerve, approximately 6 cm below the bifurcation of the carotid artery. A longitudinal incision was then made from the midpoint of the incision under microscopy, and the adventitia of the common carotid artery was bluntly dissected proximally and distally. The dissection proximity reached the bifurcation of the common carotid artery, followed by upward separation of the internal and external carotid arteries and dissection of the 1-cm-long segment, whereas the dissection of the distal region reached approximately 6 cm below the bifurcation of the common carotid artery. The surgery was performed cautiously to protect the surrounding tissues and avoid tissue damage caused by excessive stretching. Thorough hemostasis was performed after dissection of the exenterated common carotid artery. Additionally, active bleeding and significant surrounding tissue damage were recorded. Finally, the incision was closed layer by layer (Fig. 1).

### Definitions

We define premature birth as delivery after 28 weeks of pregnancy but less than 37 weeks.

The CFCS was used to characterize communication ability in children with CP. The CFCS is a reliable and valid tool for describing communication ability in children with CP [16, 17]. The CFCS ranges from level I to level V, wherein level I indicates effective sender and receiver with unfamiliar and familiar partners, and level V indicates seldom effective sender and receiver even with familiar partners. Children with CP were preoperatively evaluated for communication ability using the CFCS. The CFCS was used to re-evaluate the children’s communication ability 12 months after surgery. The children’s 12-month postoperative communication ability was compared with the preoperative communication ability. A decrease of ≥ 1 point in CFCS grade indicated a good prognosis, whereas a decrease of < 1 point indicated a poor prognosis.

The degree of salivation is assessed according to TDS. TDS is divided into five grades, with level I being the best and level V being the worst. A decrease of ≥ 1 point in TDS grade indicated a good prognosis, whereas a decrease of < 1 point indicated a poor prognosis.

Surgery is effective if one of the prognosis is good for salivation or communication ability.

The Gross Motor Function Classification System (GMFCS) was used to characterize motor function in children with CP [17]. GMFCS is divided into five levels with different grading standards based on different age groups, with level I being the best and level V being the worst. In this study, we defined GMFCS levels I–III as mild to moderate motor dysfunction and GMFCS levels IV–V as severe motor dysfunction. A decrease of ≥ 1 point in GMFCS grade indicated a good prognosis, whereas a decrease of < 1 point indicated a poor prognosis.

### Statistical analysis

SPSS software version 25 (SPSS, IBM, Chicago, USA) was used to perform statistical analysis. Quantitative data are presented as the mean ± standard deviation and compared by t-test. Qualitative data are presented as relative numbers and were compared using the *χ*^*2*^ test. Normality tests were performed using the Kolmogorov–Smirnov test, whereas quantitative data that did not conform to normal distribution and rank data were tested using the rank-sum test. It was set at *p* < 0.05 for statistical significance.

## Results

### Comparison of general information between two groups of patients

Based on the abovementioned criteria, 406 children were included in the study and divided into two groups according to the treatment they received: the CPVS and SPR groups. The CPVS group comprised 202 children (116 males and 86 females) with an average age of (7.1 ± 3.4) years. Among them, 187 CP had communication disorders, and 71 had salivation. Fifteen patients had Communication Function Classification System (CFCS) grade I, 31 had CFCS grade II, 28 had CFCS grade III, 22 had CFCS grade IV, and 106 had CFCS grade V. One hundred and thirty-one patients had Teacher Drooling Scale (TDS) grade I, 27 had TDS grade III, 29 had TDS grade III, and 15 had TDS grade IV. Ninety-three had spastic CP, 102 had mixed CP, and seven had athetotic CP. Thirteen patients had GMFCS grade I, 60 had GMFCS grade II, 32 had GMFCS grade III, 23 had GMFCS grade IV, and 74 had GMFCS grade V. The SPR group comprised 204 children (122 males and 82 females) with an average age of (6.8 ± 3.8) years. Among them, 190 CP had communication disorders, and 61 had salivation. A total of 109 patients had spastic CP, 90 had mixed CP, five had athetotic CP, 14 had CFCS grade I, 38 had CFCS grade II, 40 had CFCS grade III, 21 had CFCS grade IV, 91 had CFCS grade V, 143 had TDS grade I, 25 had TDS grade II, 27 had TDS grade III, and 9 had TDS grade IV. Ninety-six had GMFCS grade II, 58 had GMFCS grade III, 41 had GMFCS grade IV, and 9 had GMFCS grade V (Table 1).

**Table 1:** Baseline data were compared between the SPR group and the CPVS group

Factors	CPVS group (202 cases)	SPR Group (204 cases)	*χ*^*2*^*/Z*	*p* Value
Gender (numbers)				
Male	116	122		
Female	86	82	0.237	0.627
Age (years)	7.1 ± 3.4	6.8 ± 3.8	1.224	0.214
Cerebral palsy classification (numbers)				
Mixed cerebral palsy	102	90		
Spastic cerebral palsy	93	109		
Athetoid cerebral palsy	7	5	2.637	0.268
CFCS (numbers)				
Level I	15	14		
Level II	31	38		
Level III	28	40		
Level IV	26	21		
Level V	105	91	1.444	0.149
TDS (numbers)				
Level I	131	143		
Level II	27	25		
Level III	29	27		
Level IV	15	9	1.241	0.214
GMFCS (numbers)				
Level I	13	0		
Level II	60	96		
Level III	32	58		
Level IV	23	41		
Level V	74	9	4.456	0.001

### Efficacy of CPVS

**Fig. 2:**
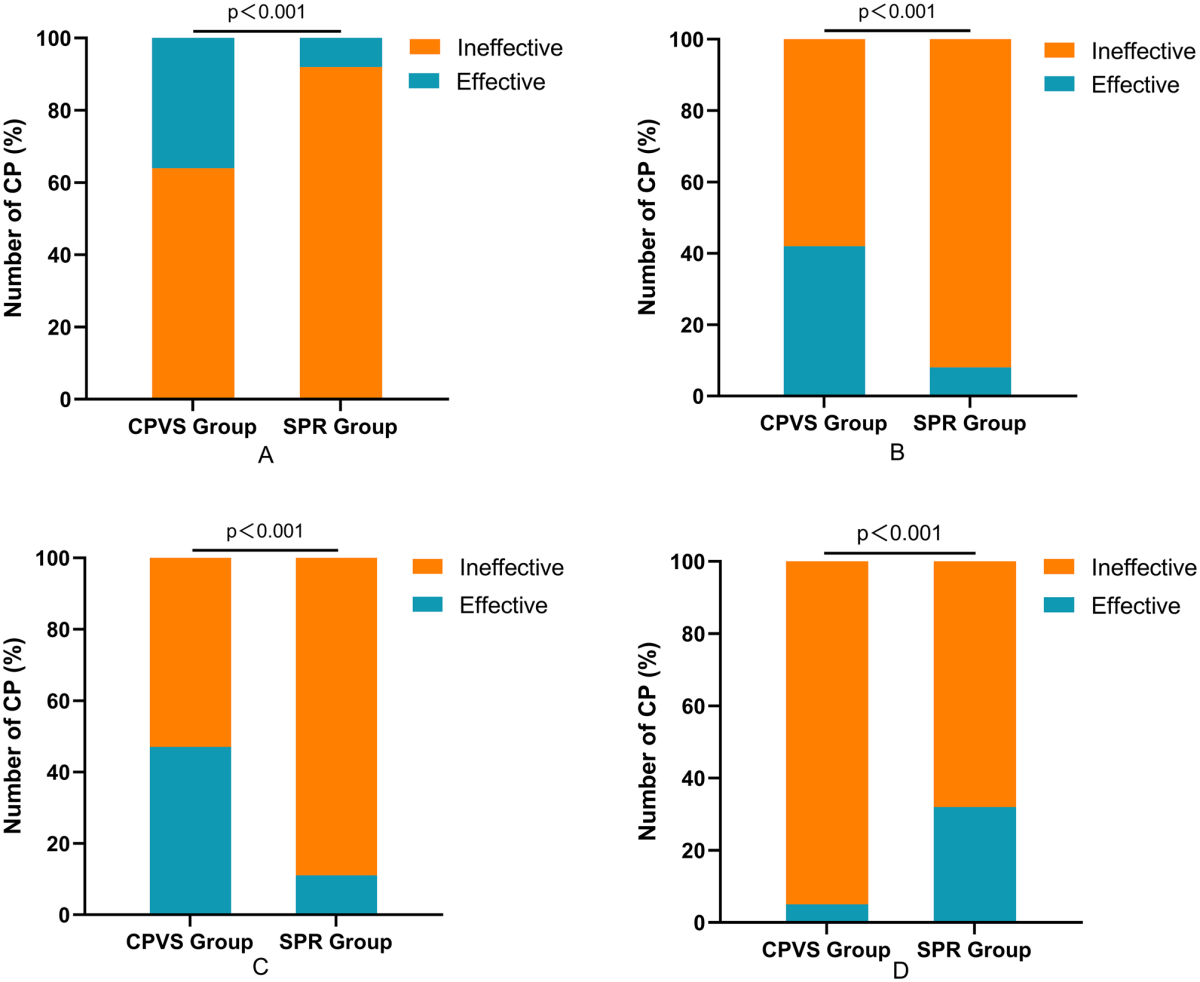
Efficacy of CPVS surgery. **A** In the CPVS group, the improvement rate of communication function was 35.82%, and the improvement rate of communication function in the SPR group was 7.89% (*χ*^*2*^ = 44.4, *p* < 0.001). **B** In the CPVS group, the improvement rate of TDS was 41.5%, and the improvement rate of TDS in the SPR group was 8.2% (*χ*^*2*^ = 18.3, *p* < 0.001). **C** In the CPVS group, the rate of surgical efficacy was 47.01%, and the irate of surgical efficacy in the SPR group was 10.78% (*χ*^*2*^ = 78.24, *p* < 0.001). **D** The motor function improvement rate was 5.29% in the CPVS group and 31.86% in the SPR group (*χ*^*2*^ = 44.8, *p* < 0.001)

There were communication disorders among 187 children in the CPVS group. Sixty-eight children with CP had a good prognosis, 119 children with CP had a poor prognosis, and the rate of improvement in language function was 36.3%. Among the 190 children in the SPR group, 15 children with CP had a good prognosis, 175 children with CP had a poor prognosis, and the effective rate of improving language function was 7.89%. Compared with the improvement rate of language function between the two groups, the CPVS group was significantly higher than that in the SPR group (*χ*^*2*^ = 44.4, *p* < 0.001), and the difference was statistically significant (Fig. 2A).

Among the 71 children with CP with drooling in the CPVS group, 29 had a good prognosis, and the improvement rate of saliva was (41.5%); Among the 61 children with CP in the SPR group, only 5 had a good prognosis, and improvement rate of saliva was (8.2%), and the CPVS group was significantly higher than that in the SPR group (*χ*^*2*^ = 18.3, *p* < 0.001), and the difference was statistically significant (Fig. 2B).

The effective rate of surgery in the CPVS group was 47.01%, which was significantly higher than that in the SPR group 10.78% (*χ*^*2*^ = 78.24, *p* < 0.001) (Fig. 2C).

In terms of motor function, the rate of motor function improvement in the CPVS group was 5.29% (10/189), while the rate of motor function improvement in the SPR group was 31.86% (65/204). The SPR group was significantly higher than the CPVS group (*χ*^*2*^ = 44.8, *p* < 0.001), and the difference was statistically significant (Fig. 2D).

### Univariate analysis of factors influencing surgical efficacy

Univariate analysis showed no significant differences in age, sex, and epilepsy between the group with a good and poor surgical prognosis. There were significant differences in GMFCS grade and preterm birth between the group with good and poor surgical prognoses (Table 2). Univariate studies with *P* ≤ 0.2 entered multivariate regression.

**Table 2:** Univariate analysis of factors influencing surgical efficacy

Factors (self-variable name)	Packet (assignment description)	Good prognosis	Poor prognosis	*χ*^*2*^*/Z*	*p* Value
Gender	Male (= 0)	56	60		
	Female (= 1)	39	47	0.17	0.68
Age	≤ 7 (= 0)	61	62		
	> 7 (= 1)	34	45	0.83	0.36
Cerebral palsy classification	Mixed cerebral palsy (= 0)	42	61		
	Spastic cerebral palsy (= 1)	49	43		
	Athetoid cerebral palsy (= 2)	4	3	3.076	0.215
GMFCS	Level I-III (= 0)	63	42		
	Level IV-V (= 1)	32	65	14.76	< 0.001
Epilepsy	No (= 0)	71	71		
	Yes (= 1)	24	36	1.69	0.19
Neonatal asphyxia	No (= 0)	54	64		
	Yes (= 1)	41	43	0.18	0.66
Preterm birth	Yes (= 0)	33	22		
	No (= 1)	62	85	2.10	0.02

### Multivariate analyses of factors influencing surgical efficacy

Logistic regression multivariate analysis showed that GMFCS grade and preterm birth were prognostic factors affecting efficacy (Table 3).

**Table 3:** Logistic regression analysis for multiple factors affecting the efficacy of CPVS

					Exp (*B*) 95% CI	
Factors	*B* value	S.E. value	*p* Value	Exp (*B*)	Lower limit	Upper limit
GMFCS	− 1.106	0.299	0.000	0.331	0.184	0.594
Epilepsy	− 0.319	0.332	0.336	0.727	0.379	1.393
Preterm birth	− 0.700	0.329	0.038	0.497	0.257	0.961

### Adverse effects of CPVS

After surgery, four patients developed subcutaneous or intramuscular hematoma (2.1%), and the hematoma disappeared 2 ~ 7 days after surgery. Fever in 15 patients (8%) disappeared 3 days after surgery. Three patients (1.6%) developed mild skin burns. One patient (0.55%) developed a persistent drinking cough.

## Discussion

CP is a common central neurological disorder in children characterized by postural abnormalities, central motor dysfunction, and communication impairment. Varying degrees of communication dysfunction and salivation are observed in approximately 33–66% of children with CP [18, 19]. Communication disorder and salivation in children with CP affect their verbal communication and adversely impact their mental health, causing fear, low self-esteem, dependence, and isolation, thereby impairing their normal physical and mental growth and socialization [7].

SPR effectively alleviates permanent spasticity in children with CP by selectively dissecting the afferent class I_a_ nerve fibers in the muscular spindle and reducing alpha neuron activity, thereby decreasing muscular tension [20]. No studies have reported that SPR improves communication disorder and salivation in children with CP. Therefore, we used children with CP undergoing SPR as a control group to exclude the influence of their growth and development on the study of children with CP.

CPVS has demonstrated some degree of efficacy in children with CP [13], consistent with the present study’s findings. In this study, the rate of communication disorder improvement in the CPVS group was 35.82%, slightly lower than in the previous study (45%) [13]. In the above study, the parents assessed the improvement in communication function in children with CP. However, the present study used the CFCS to evaluate communication function in children with CP. The variations in the results are attributable to the different criteria for efficacy evaluation. In terms of improved salivation, previous studies have found that CPVS improved by 46.9% at 4 weeks and 25% at 24 weeks [12]. The improvement rate of salivation in this study was 41.5%, the same as previous studies regarding surgical efficacy. However, in terms of efficacy trends, the results of the two studies were different, so a larger sample size and more reasonable experimental design were needed to illustrate this issue.

The rate of communication function and salivation improvement in the SPR group was 7.89% and 8.2%, which is consistent with a previous study’s findings [21]. Children with CP will have very low self-recovery ability as they grow and develop. The significant difference in the rate of communication function and salivation improvement between the two groups indicates that CPVS effectively treats communication disorder and salivation in children with CP.

The mechanism by which the efficacy of CPVS in children with CP has not been elucidated. In a previous study, single-photon emission computer tomography confirmed inadequate cerebral blood perfusion to the contralateral side of the hemiplegic limb in children with CP [22]. The brain is extremely sensitive to ischemia. Brain and nerve development depend on the perfusion of the cerebral circulation, and even transient ischemia can impair neurodevelopment [23]. Regulating cerebral blood flow largely depends on the superior cervical ganglion and sympathetic nerves around the carotid artery. Increased sympathetic stimulation leads to arterial contraction, whereas removal or anesthesia of the sympathetic nerves increases the dilatability of the small arteries [24]. Secondary changes in cerebral blood flow and cerebral vasculature after CPVS improve cerebral circulation, particularly microcirculation in the cerebral cortex, thereby improving the symptoms in children with CP. Improved cerebral circulation to promote brain and nerve development might be one of the mechanisms by which CPVS communication function in children with CP [13]. Local neuroendocrine and central neurotransmitter alterations might be caused by CPVS. An animal experiment [25] revealed that norepinephrine-like neurofibrillary groups disappeared immediately after the removal of the bilateral superior and middle cervical sympathetic ganglia in the arteries of the ring of Wills or the iris of the mouse brain. However, the neuropeptide-like neurofibrillary groups were preserved in 18–32% of the mice, which might be another mechanism by which the efficacy of CPVS in children with CP. The rate of efficacy of CPVS in children belonging to the CPVS group did not reach 100% due to the combination of various factors influencing symptoms, such as the child’s cognitive and expressive abilities [26]. Therefore, the mechanisms by which the efficacy of CPVS in children with CP need further research.

Currently, there are no studies on the influencing factors of the efficacy of CPVS. This study found that preoperative severe dyskinesia status in children with CP was an independent predictor of poor prognosis after CPVS in children with CP. Similarly, according to the GMFCS [17], children with CP with movement disorders class V do not resist gravity in the head and trunk, inability to perform the voluntary movement, and have a complete loss of exercise ability. It can be seen that such patients have extremely severe brain damage and little recovery potential. Malandraki et al. [27] found that children with unilateral spastic CP were more likely to have dysphagia and motor aphasia than normally developing children, and their performance varied greatly between patients. This result is consistent with our clinical data. Children with severe dyskinesia with CP have more severe clinical symptoms and a poorer prognosis after CPVS due to their more severe brain damage. Therefore, the results of this study show that CP with severe motor dysfunction CP has a higher incidence of poor prognosis after CPVS. Preterm birth is a serious health problem worldwide and the leading cause of death among children under five [28]. Premature infants have a reduction in overall brain volume, especially in the frontotemporal and hippocampus [16], which has more severe brain damage and more difficulty in recovery than in full-term children. Our findings suggest that preterm birth is a risk factor for poor prognosis after surgery for CPVS.

CPVS does have some effect in improving salivation and communication function, but our findings imply that CPVS should be carefully considered in future clinical work in children with CP with severe dysmotility or preterm birth, who may not necessarily benefit from surgery in addition to the trauma and financial burden of surgery. CPVS has limited effect on improving motor function and is not recommended for improving motor function in CP. SPR has some effect on improving motor function, but we found in our data that SPR is not recommended in children with GMFCS grade IV–V, because rehabilitation is not effective in these children, and surgery alone to improve limb spasticity does not improve motor function. In summary, SPR is suitable for alleviating the spasticity of children with CP, and SPR surgery is recommended for children with GMFCS grade I–III. CPVS is suitable for alleviating salivation and improving communication function in CP, and CPVS surgery is recommended for children with GMFCS grade I–III and CP without a history of preterm birth. For children with CP who meet both the indications for SPR and the CPVS, we recommend SPR therapy first to relieve limb spasticity, because SPR has been shown to be a safe and effective treatment in multiple studies, and CPVS surgery is performed 3 months after SPR. For those with motor dysfunction, and premature birth of CP, early intervention for CP should be committed to isolating the brain damage factors before the peak of brain damage, blocking the subsequent brain damage process, and providing opportunities for subsequent surgical treatment.

In previous studies, patients assessed whether the child's communication function or salivation improved after surgery. However, the present retrospective cohort study used the scale to evaluate surgical efficacy in children with CP to obtain more objective results. A controlled study matching children with CP who underwent CPVS with those undergoing SPR revealed that the growth and development of children were not responsible for the surgical efficacy brought about by CPVS. It further proves the efficacy of CPVS in children with CP and discusses the influencing factors of surgical efficacy. The present study has some limitations. First, a long-term observation of surgical efficacy in children with CP was performed. Therefore, the long-term efficacy of CPVS in children with CP needs further research. In addition, we should continue to include more features of CP with good and poor prognoses after CPVS so that we can better explore the mechanism of CPVS efficacy and individualize treatment plans for different children with cerebral palsy.

## Conclusion

CPVS is a safe and effective procedure for treating children with CP, and preterm birth and GMFCS level are independent factors of surgical efficacy. A better understanding of the factors that affect the efficacy of CPVS can help surgeons predict the efficacy before surgery to select the best treatment and improve the success rate of surgery.

## Data Availability

The data that support the findings of this study are available on request from the corresponding author, [Xinping Luan], upon reasonable request.

## Abbreviations

CP: Cerebral palsy
CPVS: Cervical perivascular sympathectomy
CFCS: Communication Function Classification System
GMFCS: Gross Motor Function Classification System
MMD: Moyamoya disease
SPR: Selective posterior rhizotomy
TDS: Teacher Drooling Scale
